# Apomorfina en Infusión Continua: Impacto Económico en España

**DOI:** 10.31083/RN38994

**Published:** 2026-02-26

**Authors:** Lina Micolta-Córdoba, Juan Pablo Castro-Ramirez, Carlos Reyes-Ortiz

**Affiliations:** ^1^Residente Geriatría, Facultad de la Salud, Universidad del Valle, 760043 Santiago de Cali, Colombia; ^2^Departamento de Clínicas Médicas, Facultad de Ciencias de la Salud, Pontificia Universidad Javeriana, 720001 Cali, Colombia; ^3^Institute of Public Health, College of Pharmacy and Pharmaceutical Sciences, Florida Agricultural and Mechanical University, Tallahassee, FL 32307, USA

La Enfermedad de Parkinson (EP) es el segundo trastorno neurodegenerativo más prevalente en el mundo después de la enfermedad de Alzheimer y su principal factor de riesgo es la edad [1]. De acuerdo con los últimos datos globales, esta patología afectó en 2021 a 11,8 millones de personas, de las cuales un 75,3% corresponden a ≥65 años o personas mayores (PM). Debido a su naturaleza crónica y progresiva representa un importante desafío para la salud pública, demostrado por la alta carga de enfermedad que ocasiona, generando 7,5 millones de años de vida ajustados por discapacidad (AVAD) (85,2% en PM) y aumentando los costos para los sistemas de salud, la sociedad y los propios pacientes con sus familias y cuidadores [2]. Costos que alcanzan su máximo en los estadios graves y que condicionan un descenso en espejo de la calidad de vida [1]. Patrón replicado en Europa, donde en 2021 generó 1,5 millones de AVAD (92,1% en PM) y en España, donde causó 105.166 AVAD (93,6% en PM) [2]. País que para 2023 alcanzó un índice de envejecimiento elevado (137,7%), explicado por una población >10 millones de PM (24,3% del total de habitantes), con estimaciones para 2035 de alcanzar 14,8 millones. El estallido demográfico ha ido de la mano con el incremento de la prevalencia e incidencia de la EP (5,6% y 5,0% entre 2011 y 2021) y de la dependencia funcional (2,6 millones de PM con discapacidad en 2020) [2, 3].

A pesar de disponer de varias terapias farmacológicas, el pilar del manejo continúa siendo la levodopa. Sin embargo, con la progresión de la neurodegeneración su uso se asocia a discinesias y fluctuaciones motoras y no-motoras, explicadas en parte por alteraciones en la deglución o motilidad gastrointestinal que llevan a niveles inestables del fármaco en sangre, a regímenes orales complejos de múltiples dosis y aún más importante, al deterioro de la calidad de vida [4, 5]. Por ello, en la fase avanzada de la enfermedad, se opta por las terapias asistidas por dispositivos (TAD), como la estimulación cerebral profunda (ECP), la infusión intrayeyunal de levodopa/carbidopa +/- entacapona en gel intestinal (LCGI) y algunas estrategias menos invasivas y totalmente reversibles, como la infusión subcutánea continua de foslevodopa/foscarbidopa o de apomorfina (ISCA); cada una con un beneficio clínico demostrado en ensayos clínicos, ninguno cabeza a cabeza, pero con una implementación subóptima debido a su alto costo [4].

En relación con esta última TAD, García-Fernández *et al*. [6] publicaron una serie de casos de España, con una alta proporción de PM (edad promedio: 70,8 años), en la que la ISCA extendida a la noche, al evitar una estimulación pulsátil no-fisiológica de los receptores dopaminérgicos, demostró beneficios no solo en los síntomas motores sino en los no-motores como la calidad del sueño, a costa de una alta tasa de interrupción del tratamiento (43,2%), particularmente por falta de adaptación al dispositivo o reacciones adversas. Situación confirmada en una revisión sistemática de 82 estudios [7], que reportó que las reacciones adversas son muy frecuentes (hasta en el 57%, especialmente durante los primeros meses) y que el manejo del dispositivo puede convertirse en un desafío técnico que sobrecarga al cuidador, a pesar del beneficio demostrado en la calidad de vida [5]. Considerando que el mejor perfil de pacientes para esta terapia sería aquellos con edad avanzada y buena respuesta previa a levodopa, sin discinesias bifásicas, pero que presenten síndromes geriátricos como: trastornos de la marcha o inestabilidad postural en estado “*on*” y caídas, deterioro cognitivo no-severo, depresión o trastornos del sueño que no sean candidatos a la ECP o como terapia puente a esta y que no tengan contraindicaciones absolutas: insuficiencia hepática, hipotensión ortostática o psicosis refractarias [4, 7, 8].

Es entonces cuando se requiere de una evaluación económica rigurosa para guiar la toma de decisiones en cuanto a las políticas de implementación de la ISCA en un país con una demanda creciente y unos recursos sanitarios limitados, como España y que a su vez, no esté sesgada por la financiación de la industria farmacéutica, como se ha descrito en varios estudios económicos de TAD [1]. Con este objetivo se realizó un análisis de costo-efectividad actualizado a diciembre de 2024 y sustentado en el marco del costo de oportunidad en salud. Para esto, se empleó la metodología descrita por Vivancos-Matellano *et al*. [8] para actualizar los costos directos sanitarios, desde la perspectiva del Sistema Nacional de Salud (SNS) de España, asumiendo una tasa de descuento del 3,5% y teniendo en cuenta el Índice de Precios de Consumo del grupo de bienes y servicios “Sanidad o Medicina” [9], obteniendo los siguientes costos totales a 5 años:

∙ ISCA: €100.672,962 (promedio anual: €20.134,045) (1 EUR = 1,05 USD).

∙ LCGI: €214.070,085 (promedio anual: €42.813,470).

∙ ECP: €81.631,879 (promedio anual: €16.326,011).

Se continuó con una evaluación de costo-utilidad, fundamentada en los años de vida ajustados por calidad (AVAC) estimados previamente por modelos de Markov que asumen que la mitad de los pacientes se encontraban en estado “*off*” >14 h/día al inicio del tratamiento [8]:

∙ ISCA: 34.895,307 €/AVAC.

∙ LCGI: 68.612,207 €/AVAC.

∙ ECP: 29.154,242 €/AVAC.

De esta forma, se concluye que la LCGI es la opción terapéutica más costosa y con la relación costo-utilidad menos favorable. En contraste, la ECP se posiciona como la alternativa más asequible y con el mejor perfil de costo-utilidad. Finalmente, al comparar los costos con el umbral de costo-efectividad de 27.000–34.000 €/AVAC, según la actualización más reciente [10], se demuestra que la ECP es, actualmente, la única opción costo-efectiva, relegando a la ISCA a un rango limítrofe, que probablemente se vuelva costo-efectivo en los próximos años debido a la tasa de descuento anual y la reducción en el precio de sus insumos.

Si bien esta evaluación económica presenta limitaciones, como la cuantificación de la utilidad basada únicamente en los síntomas motores y la falta de medición de los costos directos no sanitarios e indirectos; sigue siendo, hasta el momento, la mejor herramienta disponible para respaldar la implementación de estas terapias en España. En particular, destaca el impacto positivo, tanto clínico como económico, del uso de apomorfina en las PM con síndromes geriátricos.

## Contribuciones de los Autores

LMC: Conceptualización, Curación de datos, Análisis formal, Investigación, Administración del proyecto, Redacción – borrador original, Validación, Visualización. JPCR: Curación de datos, Análisis formal, Investigación, Redacción – revisión y edición, Supervisión, Validación, Visualización. CRO: Validación, Visualización, Redacción – revisión y edición. Todos los autores leyeron y aprobaron el manuscrito final. Todos los autores han participado lo suficiente en el trabajo y han acordado ser responsables de todos los aspectos del mismo.

## Aprobación Ética y Consentimiento Informado

No aplicable.

## Financiación

Esta investigación no recibió financiación externa.
